# The PKC/NOX/ROS and PYK2/MEK/ERK/PARP signalling pathways drive TRPM2 channel activation induced by non-cytolytic oxidative stress in microglial cells

**DOI:** 10.1080/13510002.2025.2503131

**Published:** 2025-05-15

**Authors:** Sharifah Alawieyah Syed Mortadza, Nur Zulaikha Mohamad Zahir, Chew Tze Wei, Lin-Hua Jiang

**Affiliations:** a Sino-UK Joint Laboratory of Brain Function and Injury of Henan Province, Department of Physiology and Pathophysiology, Xinxiang Medical University, Xinxiang, People's Republic of China; b Department of Biochemistry, Faculty of Biotechnology and Biomolecular Sciences, Universiti Putra Malaysia, Serdang, Malaysia; c School of Biomedical Sciences, Faculty of Biological Sciences, University of Leeds, Leeds, UK

**Keywords:** Oxidative stress, TRPM2, PKC, NOX, PYK2, ERK, microglia, PARP-1

## Abstract

**Objectives:**

The study aimed to investigate the signalling mechanism for TRPM2 channel activation by non-cytolytic oxidative stress in microglia.

**Methods:**

Microglia from wild-type (WT) and TRPM2-knockout (KO) mice were exposed to 10-30 mM H_2_O_2_ for up to 24 hours. Morphological changes characteristic of microglial activation, [Ca^2+^]_c_, ROS generation and the effects of inhibiting particular signalling pathways were examined.

**Results:**

Exposure of WT microglia to H_2_O_2_ for 24 hours caused no cell death but induced salient morphological changes, which was prevented by TRPM2-KO. Exposure of WT microglia to H_2_O_2_ to 2 hours failed, and extension to 8 hours was required, to induce an increase in [Ca^2+^]_c_, which was abolished by TRPM2-KO. Exposure of microglia to H_2_O_2_ for 8 hours induced ROS generation, which was suppressed by inhibition of PKC and NADPH oxidases (NOX). H_2_O_2_-induced PARP activation in TRPM2-KO cells was lower than that in WT cells. Furthermore, H_2_O_2_-induced activation of PARP and TRPM2 and morphological changes were attenuated by inhibition of PCK and NOX as well as PYK2 and MEK/ERK.

**Conclusion:**

Our results support that PKC/NOX-mediated ROS generation and TRPM2-mediated Ca^2+^-induced activation of the PYK2/MEK/ERK pathway form a positive feedback mechanism to drive TRPM2 channel activation by non-cytolytic oxidative stress.

## 1. Introduction

Microglia are the major type of immune cells residing in the central nervous system (CNS) and, under normal conditions, they continuously patrol the surrounding environments to maintain the CNS homeostasis. In response to CNS injury or insults, microglial cells transform from the homeostatic state to the activated state, which is accompanied by salient changes from branched/ramified morphology to ameboid form [1,2]. Aberrant microglial activation results in excessive production of proinflammatory mediators, including reactive oxygen species (ROS), and such microglial cell-mediated neuroinflammation plays a vital role in the pathogenesis and progression of multiple CNS damage and disease conditions [2,3].

ROS are a group of oxygen derived and chemically highly active molecules. Under physiological conditions, mammalian cells produce a small amount of ROS, which are timely removed by the antioxidant capacity of cells. A low level of ROS can act as signalling molecules to regulate cell proliferation and other functions [4]. However, under pathological or disease conditions, cells are directed to produce excessive ROS and/or their antioxidant defence is compromised, leading accumulation of high levels of ROS to induce oxidative stress [5,6]. It has been well documented that oxidative stress can cause oxidative damage by eliciting cell death under diverse CNS damage and disease conditions. However, oxidative stress, occurring under CNS damage and disease conditions, induces morphological changes and microglial activation that provides a neuroprotective role in the early stage, but chronic and low grade of microglial cell-mediated inflammation causes neuronal damage and accelerate disease progression [5–7].

Transient receptor potential melastatin-related 2 (TRPM2) is a Ca^2+^-permeable cation channel [8] and widely distributed in immune cells, including microglial cells in the CNS [9,10]. The TRPM2 channel is gated by binding of intracellular ADP-ribose (ADPR) and can be potently, albeit indirectly, activated by ROS or oxidative stress that promotes generation of ADPR mainly via the poly(ADPR) (PAR) polymerase (PARP)-mediated DNA repair mechanism in the nucleus [11–13] and also via NADase in the mitochondria [14]. Whether other signalling mechanisms are engaged in ROS-induced TRPM2 channel activation remains less defined. TRPM2-mediated Ca^2+^ influx, as shown in monocytes, activates Ca^2+^-sensitive proline-rich tyrosine kinase (PYK2) and downstream MEK/ERK signalling pathway to promote cytokine expression [15]. There is evidence that the MEK/ERK signalling plays a role in ROS-induced activation of PARP-1, the major PARP isoform in the nucleus [16]. Our previous study showed that severe oxidative stress induced by exposure to high levels of H_2_O_2_ or Zn^2+^ caused microglial cell death by activating protein kinase C (PKC) and NADPH oxidases (NOX) to generate ROS and, in addition, TRPM2-mediated Ca^2+^ influx triggers the PYK2/MEK/ERK signalling as a positive feedback mechanism to facilitate the activation of PARP and TRPM2 [17]. In addition to cell death, increasing evidence support the importance of the TRPM2 channel in regulating multiple other cell functions. We performed this study to understand whether the same mechanisms drive TRPM2 channel activation to induce microglial activation by non-cytolytic oxidative stress as those described for demise of microglial cells by cytolytic oxidative stress [17]. Our results show exposure to non-cytolytic oxidative stress can induce microglial activation, depending upon the TRPM2 channel activation. Furthermore, PKC/NOX-mediated ROS generation induces initial activation of PARP and TRPM2 channel, and TRPM2-mediated Ca^2+^ influx mobilizes the PYK2/MEK/ERK signalling pathway to sustain the activity of PATRP and TRPM2 channel. Such a positive feedback mechanism is also critical for morphological changes of microglial cells indicative of microglial cell activation. Thus, our findings enrich the understanding of the molecular and signalling mechanisms mediating oxidative stress-induced activation of the TRPM2 channel and its role in redox regulation of microglial functions in pathophysiological conditions.

## 2. Materials and methods

### 2.1. Chemicals and reagents

All chemicals or reagents were commercially obtained from Sigma-Aldrich unless specified otherwise. PJ-34 was from Santa Cruz, GKT137831 and U0126 from Cayman Chemical, and chelerythrine chloride (CTC) and PF431396 from Tocris. Dimethyl sulfoxide (DMSO) was used to prepare stock solutions of the inhibitors: 2-APB (100 mM), PJ-34 (10 mM), CTC (15 mM), GKT137831 (5 mM), diphenyleneiodonium (DPI; 10 mM), apocynin (1 M), U0126 (10 mM) and PF431396 (10 mM).

### 2.2. Microglial cell preparations

Microglial cells were isolated from 1-3-day-old WT and TRPM2-KO mice following the methods described in our previous study [17]. The TRPM2-KO mice were generated previously [18]. For live cell imaging, cells were seeded in 96-well plates (Costar) at a density of 1.1–2.75 × 10^5^ cells/ml. For immunofluorescent imaging, cells were plated on poly-L-lysine-coated coverslips at 5 × 10^4^ cells/ml in 24-well plates (Costar). Cells were maintained in high-glucose DMEM supplemented with 10% foetal bovine serum, 10 units/ml penicillin and 100 µg/ml streptomycin at 37°C in a 5% CO_2_ humidified atmosphere for 48 h prior to use.

### 2.3. PI staining

Cell death was estimated by propidium iodide (PI) staining as described in our previous study [17]. Cells in 96-wells plates were exposed to H_2_O_2_ at the indicated concentrations for up to 24 h and then co-stained by 2 μg/ml PI and 5 μg/ml Hoechst 33342 for 30 min. Fluorescence images were captured using an Olympus IX51 fluorescence microscope equipped with a digital camera and Cell^F^ software (Olympus). Seventy-five cells per well and 225 cells in total from 3 wells for every condition were analysed using ImageJ to derive the percentage of PI-positive death cell in each independent experiment, and 3 independent experiments were performed.

### 2.4. Analysis of cell morphology

Cells were exposed to H_2_O_2_ at the specified concentrations for 24 h. In experiments examining the effects of inhibitors, cells were treated at 37°C with the inhibitor at the indicated concentration, 30 min prior to and during exposure to H_2_O_2_. In all experiments, the inhibitor with the final concentrations indicated in the figure legend, or DMSO at the lowest dilution used to dilute the inhibitors in a particular set of experiments (no more than 0.1% in any case) as solvent control, was added into the culture medium. Images were captured using an Incucyte imaging system (Sartorius) or using an EVOS Cell Imaging System (Thermo Fisher Scientific). Cell morphology was analysed using computer-assisted measurements of the form factor and aspect ratio, as described in a previous study [19]. The form factor was calculated using the formula 4π × area/perimeter², where a value of 1.0 represents a circle, and a value closer to 0 indicates an elongated shape. The aspect ratio was defined as the length-to-width ratio, with a minimum value of 1.0 representing a circle. Seventy-five individual cells per well and 225 cells in total from 3 wells were analysed for every condition in each independent experiment, and 3–4 independent experiments were performed as indicated in the figure legend.

### 2.5. Single-cell Ca^2+^ imaging

Cells seeded in 96-well plates were exposed to H_2_O_2_ at the specified concentrations for 2 or 8 h. After that, cells were loaded with 5 µg/ml Fluo4/AM (Life Technologies) in standard bath solution (SBS; 134 mM NaCl, 5 mM KCl, 0.6 mM MgCl_2_, 1.5 mM CaCl_2_, 8 mM glucose, and 10 mM HEPES, pH 7.4) at room temperature for 45 min and, upon addition of Hoechst with the final concentration of 5 µg/ml, left at room temperature for further 15 min. In experiments examining Ca^2+^ influx, Ca^2+^-free SBS (134 mM NaCl, 5 mM KCl, 0.6 mM MgCl_2_, 0.4 mM EDTA, 8 mM glucose, and 10 mM HEPES, pH 7.4) was used. For experiments examining the effects of inhibitors, Fluo4/AM-loaded cells were treated at 37°C with the inhibitor at the indicated concentrations or DMSO (0.067% (v/v) for 1:1500 being the lowest dilution used to dilute the inhibitor), 30 min prior to and during exposure to H_2_O_2_. Images were captured using an EVOS Cell Imaging System. The Fluo4 fluorescence intensity in each cell was measured and the background fluorescence intensity from the same image was subtracted, using ImageJ. Fifty individual cells per well and 150 cells in total from 3 wells were examined for every condition in each independent experiment, and 3 independent experiments were performed.

### 2.6. Measurement of ROS production

Cellular ROS production was measured using 2’,7’-dichlorodihydrofluorescein diacetate (DCFH-DA) as described previously [17]. Briefly, cells seeded in 96-well plates were firstly exposed to H_2_O_2_ at the specified concentrations for 8 h. Cells were washed with SBS and incubated with 20 µM DCFH-DA in SBS at 37°C for 30 min. Following DCFH-DA staining, cells were counterstained with 5 µg/ml Hoechst at room temperature for further 15 min. For experiments examining the effects of inhibitors, Fluo4/AM-loaded cells were treated at 37°C with the inhibitor at the indicated concentrations or DMSO (0.067%(v/v)), 30 min prior to and during exposure to H_2_O_2_. Images were captured using an EVOS Cell Imaging System. The DCF fluorescence intensity of each cell was quantified, and the background fluorescence intensity from the same image was subtracted using ImageJ. Fifty individual cells per well and 150 cells in total from 3 wells were examined for every condition in each independent experiment, and 3 independent experiments were performed.

### 2.7. Immunofluorescent staining

Cells were exposed to H_2_O_2_ at the indicated concentrations for 8 h before being fixed with 4% paraformaldehyde in deionized water for 15 min and permeabilized using phosphate-buffered saline (PBS) containing 0.1% Triton X-100. After rinsing with PBS containing 0.5% Tween-20 (PSBT), cells were blocked in PBS containing 5% goat serum for 30 min, and then incubated overnight at room temperature with primary mouse anti-PAR antibody (Enzo) at a dilution of 1:500. Following extensive washing with PSBT, cells were treated with secondary fluorescein isothiocyanate-conjugated anti-mouse IgG antibody (Sigma; 1:1000) for 1 h at room temperature. After being washed with PBS and rinsed with water, the coverslips were mounted using a fluorescent mounting reagent containing 4’,6-diamidino-2-phenylindole (DAPI) (Life Technologies). For experiments examining the effects of inhibitors, cells were treated at 37°C with the inhibitor at the indicated concentrations or DMSO (0.067%(v/v)), 30 min prior to and during exposure to H_2_O_2_. Images were captured with an EVOS Cell Imaging System. The fluorescence intensity of each cell was quantified and the background fluorescence intensity from the same image was subtracted using ImageJ. Fifty individual cells per well and 150 cells in total from 3 wells of cells were examined for every condition in each independent experiment, and 3 independent experiments were performed.

### 2.8. Data presentation and statistical analysis

Data are presented as mean ± standard error of the mean (SEM), calculated from 3 to 4 average values, with each average value from each independent experiment, and N indicates the number of independent experiments. Statistical analysis was performed using Student’s *t*-test for comparisons between two groups and one-way ANOVA followed by Tukey’s post hoc test for comparisons among multiple groups. A *p*-value of less than 0.05 (*p* < 0.05) was considered statistically significant.

## 3. Results

### 3.1. Exposure to non-cytolytic oxidative stress induces trpm2-dependent morphological changes in microglial cells

**Figure 1.**
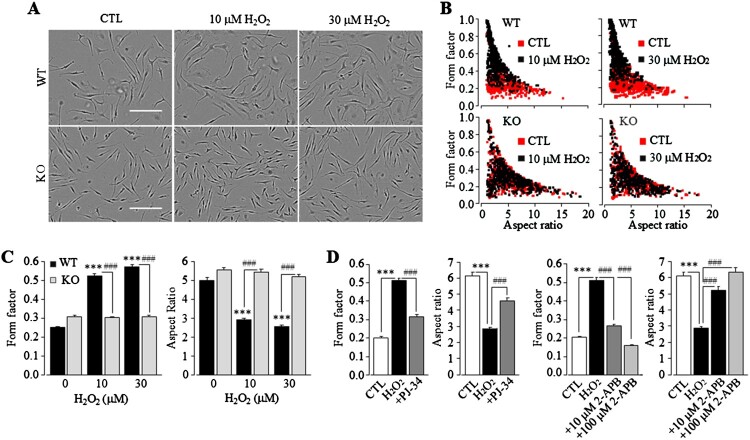
Exposure of microglial cells to non-cytolytic oxidative stress evokes TRPM2-mediated morphological changes. (A) Representative phase-contrast images showing the morphology of WT and TRPM2-KO cells without (CTL) or with exposure to H_2_O_2_ at indicated concentrations for 24 h, imaged using an Incucyte imaging system. Scale bar, 50 µm. (B and C) Scatter plots showing the distribution of form factor and aspect ratio values of individual cells (B), and mean form factor and aspect ratio values of cells under indicated conditions (C). (D) Mean form factor and aspect ratio values of cells after exposure to 10 µM H_2_O_2_ for 24 h. Cells were prior treated with 1 μM PJ-34 or 10 or 100 μM 2-APB or DMSO as solvent control, 30 min prior to and duration exposure to H_2_O_2_. The mean values represent mean ± SEM of 4 average values from *N* = 4 independent experiments (C) or 3 average values from *N* = 3 independent experiments (D), with each average value from each independent experiment analysing 225 cells in 3 wells (75 cells per well) for every condition. ***, *p* < 0.005 compared to cells without exposure to H_2_O_2_ (CTL). ###, *p* < 0.005 compared to WT cells exposed to the same concentrations of H_2_O_2_ (C) or H_2_O_2_-exposed cells prior treated with DMSO as solvent control (D).

**Figure 2.**
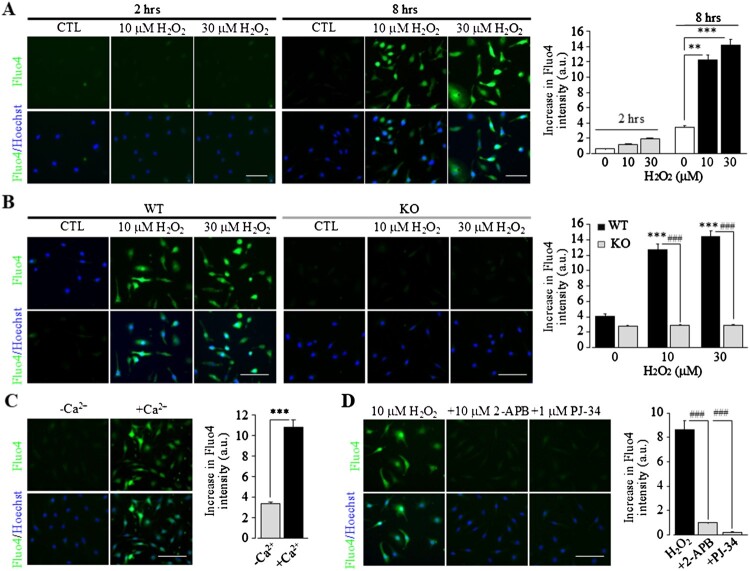
Extended exposure is required for TRPM2 channel activation by non-cytolytic oxidative stress in microglial cells. (A) *Left*, representative images showing Ca^2+^ responses (top: Fluo4; bottom: co-staining with Hoechst) of individual WT cells induced by exposure to indicated concentrations of H_2_O_2_ for 2 or 8 h in Ca^2+^-containing solutions. *Right*, mean H_2_O_2_-induced Ca^2+^ responses under indicated conditions. (B) *Left*, representative images showing Ca^2+^ responses of individual WT or TRPM2-KO cells induced by exposure to indicated concentrations of H_2_O_2_ for 8 h in Ca^2+^-containing solutions. *Right*, mean H_2_O_2_-induced Ca^2+^ responses under indicated conditions. (C) *Left*, representative images showing Ca^2+^ responses of individual WT cells induced by exposure to 10 µM H_2_O_2_ for 8 h in Ca^2+^-free or Ca^2+^-containing solutions. *Right*, mean H_2_O_2_-induced Ca^2+^ responses under indicated conditions. (D) *Left*, representative images showing Ca^2+^ responses of individual WT cells induced by exposure to 10 µM H_2_O_2_ for 8 h in Ca^2+^-containing solutions. Cells were prior treated with 10 µM 2-APB or 1 µM PJ-34 or DMSO as solvent control, 30 min prior to and duration exposure to H_2_O_2_. *Right*, mean H_2_O_2_-induced Ca^2+^ responses under indicated conditions. The mean values (A–D) represent mean ± SEM of 3 average values from *N* = 3 independent experiments, with each average value from each independent experiment analysing 150 cells in 3 wells (50 cells per well) for every condition. Scale bar, 40 μm. *, *p* < 0.05; ***, *p* < 0.005 compared to cells without exposure to H_2_O_2_ (A,B) or cells in Ca^2+^-free solutions (C). ###, *p* < 0.005 compared to WT cells exposed to the same concentrations of H_2_O_2_ (B) or H_2_O_2_-exposed cells prior treated with DMSO as solvent control (D).

Plentiful evidence exists that oxidative stress can induce salient morphological changes characteristic of microglial activation from the homeostatic state [2,10]. To examine whether the TRPM2 channel mediates microglial activation or redox regulation of microglial function by non-cytolytic oxidative stress, we started with PI staining to examine cell death. Exposure of mouse microglial cells to 10 or 30 μM H_2_O_2_ for 4, 8 and 24 h resulted in no significant increase in the percentage of PI-positive dead cells as compared to that in cells without exposure to H_2_O_2_, indicating no cell death (Fig.1S). Next, we analysed the morphology of microglial cells in culture under the control condition. A majority of microglial cells displayed a rod-like morphology (Figure 1(A)), mainly in the homeostatic-like state. To quantitatively evaluate cell morphology, we performed a computer-assisted analysis of the form factor (or circularity) and aspect ratio. Both parameters exhibited widespread distribution (Figure 1(B)), indicating heterogeneity in cell morphology. Scatter plot analysis reveals no significant difference in the morphology between WT and TRPM2-KO microglia cells (Figure 1(C)). Exposure to non-cytolytic oxidative stress induced by 10 or 30 μM H_2_O_2_ for 24 h induced WT microglial cells to undergo noticeable changes in cell morphology with enlarged cell body and shortened processes and, by contrast, such changes were not observed in TRPM2-KO microglial cells (Figure 1(A–C)). Consistently, H_2_O_2_-induced change in the morphology of WT microglial cells was reduced or largely prevented by prior treatment with 10 μM or 100 μM 2-APB, respectively, which is known to inhibit the TRPM2 channel, or 1 μM PJ-34 to inhibit PARP, whose activity is required for ROS-induced ADPR generation for activating the TRPM2 channel (Figure 1(D) and Fig.2S). Taken together, these results show that exposure of microglial cells to non-cytolytic oxidative stress can induce changes in cell morphology featuring microglial activation via the TRPM2 channel.

### 3.2. Extended exposure to non-cytolytic oxidative stress is required for TRPM2 channel activation in microglial cells

**Figure 3.**
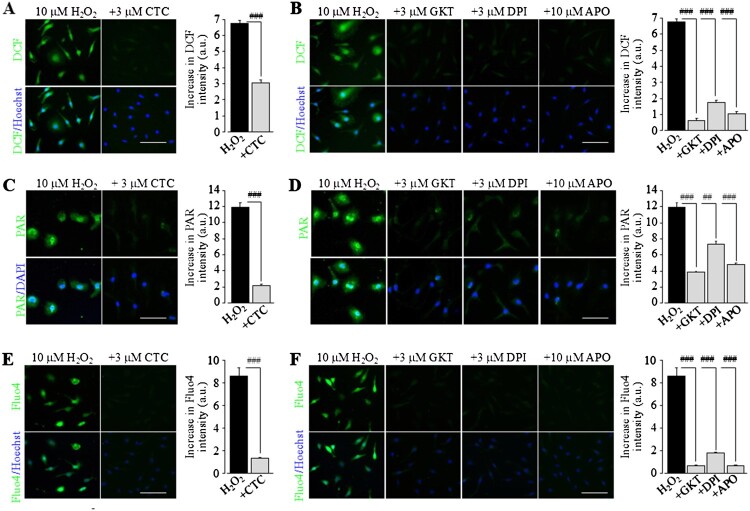
PCK/NOX-mediated ROS generation amplifies TRPM2 channel activation induced by non-cytolytic oxidative stress in microglial cells. (A and B) *Left*, representative fluorescent images showing cellular ROS level (top: DCF; bottom: co-staining with Hoechst) in individual WT cells after exposure to 10 µM H_2_O_2_ for 8 h. Cells were prior treated with 3 µM chelerythrine chloride (CTC) (A), 3 µM GKT137831 (GKT), 3 µM diphenyleneiodonium (DPI), or 10 µM apocynin (APO) (B), or DMSO as solvent control, 30 min prior to and duration exposure to H_2_O_2_. *Right*, mean ROS generation under indicated conditions. (C and D) *Left*, representative images showing PAR generation (top row: PAR; bottom row: co-staining with DAPI) in individual WT cells after exposure to 10 µM H_2_O_2_ for 8 h. Cells were treated with CTC (C), GKT, DPI, APO (D) or DMSO, 30 min prior to and during exposure to H_2_O_2_ as shown in A and B. *Right*, mean H_2_O_2_-induced PAR generation under indicated conditions. (E and F) *Left*, representative single-cell images showing Ca^2+^ responses (top: Fluo-4 fluorescence; bottom: co-staining with Hoechst) of individual WT cells after exposure to 10 µM H_2_O_2_ for 8 h. Cells were treated with CTC (E), GKT, DPI or APO (F) or DMSO as solvent control, 30 min prior to and during exposure to H_2_O_2_. *Right*, mean H_2_O_2_-induced Ca^2+^ responses under indicated conditions. The mean values (A–F) represent mean ± SEM of 3 average values from *N* = 3 independent experiments, with each average value from each independent experiment analysing 150 cells in 3 wells (50 cells per well) for every condition. Scale bar, 40 μm. ###, *p* < 0.005 compared to H_2_O_2_-exposed cells prior treated with DMSO as solvent control.

TRPM2 has been well demonstrated as a plasma membrane Ca^2+^-permeable channel in microglial cells that mediate oxidative stress-induced Ca^2+^ influx [15,20]. To directly demonstrate whether non-cytolytic oxidative stress can induce TRPM2 channel activation, TRPM2-mediated Ca^2+^ responses in microglial cells were monitored using single-cell imaging of the intensity of the fluorescent Ca^2+^ indicator Fluo4, prior loaded into cells. Exposure of WT microglial cells to 10-30 μM H_2_O_2_ for 2 h induced no or negligible increases in the [Ca^2+^]_c_, and extending the exposure duration to 8 h resulted in strong Ca^2+^ response to H_2_O_2_ (Figure 2(A)), indicating that extended exposure is necessary for inducing TRPM2 channel activation by non-cytolytic oxidative stress. In stark contrast with WT microglial cells, exposure of TRPM2-KO microglial cells to 10-30 μM H_2_O_2_ even after 8 h elicited minimal Ca^2+^ response (Figure 2(B)), unequivocally showing that H_2_O_2_-induced Ca^2+^ response is mediated by the TRPM2 channel. In addition, the Ca^2+^ response in WT microglial cells was lost when cells were bathed extracellular Ca^2+^-free solutions (Figure 2(C)), confirming the TRPM2 channel being a Ca^2+^-permeable channel in the plasma membrane [21,22]. This notion was further supported by the results that the Ca^2+^ response in WT microglial cells was strongly inhibited by prior treatment with 2-APB (Figure 2(D)). Moreover, the Ca^2+^ response was largely abolished by prior treatment with PJ-34 (Figure 2(D)). Consistently, exposure to H_2_O_2_-induced PAR generation that exhibited strong co-localization with DAPI nuclear staining (Fig. 3S), indicating activation of PARP in the nucleus. Collectively, these results confirm the well-established molecular mechanism for oxidative stress-induced TRPM2 channel activation, namely, activation of nuclear PARP to generate ADPR that in turn activates the TRPM2 channel on the cell surface to mediate extracellular Ca^2+^ influx [9,17]. However, extended exposure to non-cytolytic oxidative stress was required to induce TRPM2 channel activation (Figure 2(A)), suggesting engagement of additional molecular and signalling mechanisms in microglial cells.

### 3.3. PKC/NOX-mediated ROS generation amplifies TRPM2 channel activation by extended exposure to non-cytolytic oxidative stress

**Figure 4.**
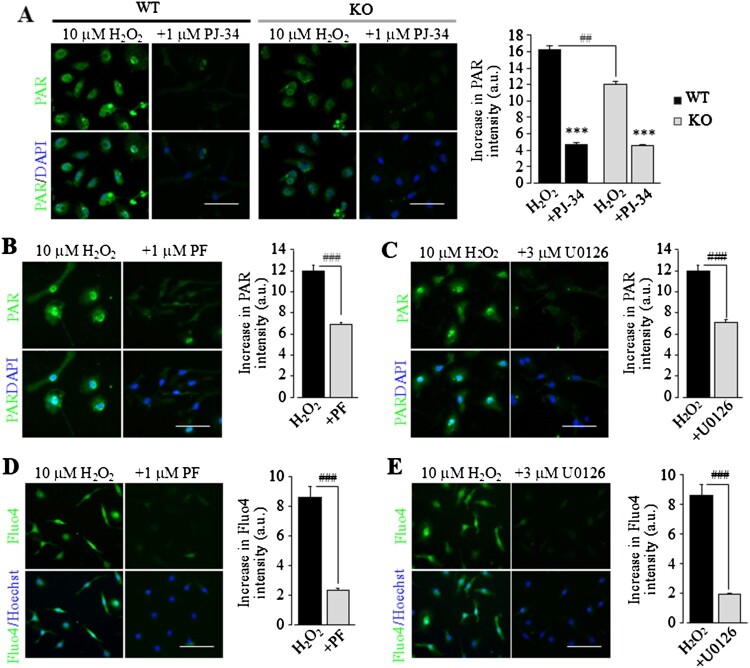
The PYK2/MEK/ERK signalling pathway facilitates TRPM2 channel activation by non-cytolytic oxidative stress in microglial cells. (A) *Left*, representative fluorescent images showing PAR formation (top: PAR; bottom: co-staining with DAPI) in individual WT and TRPM2-KO cells after exposure to 10 µM H_2_O_2_ for 8 h. Cells were treated with 1 μM PJ-34 or DMSO as solvent control, 30 min prior to and during exposure to H_2_O_2_. *Right*, mean PAR generation under indicated conditions. ***, *p* < 0.005 compared to WT or TRPM2-KO cells exposed to H_2_O_2_ and prior treated with DMSO. ##, *p* < 0.01 compared between WT and TRPM2-KO cells under the same treatment. (B and C) *Left*, representative fluorescence images showing PAR generation in individual WT cells exposed for 8 h to 10 µM H_2_O_2_. Cells were treated with 1 μM PF431396 (PF) (B) or 3 µM U0126 (C) or DMSO as solvent control, 30 min prior and duration exposure to H_2_O_2_. *Right*, mean PAR generation under indicated conditions. (D and E) *Left*, representative fluorescence images showing Ca^2+^ responses (top: Fluo4; bottom: co-staining with Hoechst) in individual cells exposed to 10 µM H_2_O_2_ for 8 h. Cells were treated with 1 μM PF (D) or 3 µM U0126 (E) or DMSO as solvent control, 30 min prior to and duration exposure to H_2_O_2_. *Right*, mean H_2_O_2_-induced Ca^2+^ responses under indicated conditions. The mean values (A–E) represent mean ± SEM of 3 average values from *N* = 3 independent experiments, with each average value from each independent experiment analysing 150 cells in 3 wells (50 cells per well) for every condition. Scale bar, 40 μm. ###, *p* < 0.005 compared to to H_2_O_2_-exposed cells prior treated with DMSO as solvent control.

ROS generation is an important factor contributing to and also amplifying oxidative stress as a critical part in the pathogenesis of multiple CNS damage and diseases [21,23]. To examine whether ROS generation is involved in TRPM2 channel activation in microglial cells following exposure to non-cytolytic oxidative stress, we monitored cellular ROS generation by measuring the intensity of the fluorescent ROS indicator DCF, prior loaded into cells. There was a remarkable increase after exposure to 10–30 μM H_2_O_2_ for 8 h (Fig. 4S), indicating that exposure to non-cytolytic oxidative stress can prompt ROS generation in microglial cells.

NOX are a group of membrane-integrated enzymes particularly used by immune cells as a critical mechanism for ROS generation, and, in addition, PKC can activate NOX [24]. Our previous studies showed that PKC/NOX-mediated ROS generation drives cell death in microglial cells by exposure to Zn^2+^ or in neurons by exposure to amyloid-42 peptide [17,25]. Thus, we asked whether PKC/NOX mediates ROS generation and TRPM2 channel activation under non-cytolytic oxidative stress. The ROS generation induced by exposure to H_2_O_2_ (10 μM) for 8 h was strongly inhibited by treatment with 3 μM CTC, a PKC blocker (Figure 3(A)), and 3 μM GKT137831, 3 μM DPI or 3 μM apocynin, three different NOX blockers (Figure 3(B)). Moreover, H_2_O_2_-induced PAR generation (Figure 3(C,D)) and Ca^2+^ responses (Figure 3(E,F)) were strongly suppressed or prevented by inhibiting PKC or NOX. These results in combination support the importance of PKC/NOX-mediated ROS generation in the activation of PARP and TRPM2 channel induced by extended exposure to non-cytolytic oxidative stress.

### 3.4. The PYK2/MEK/ERK pathway is mobilized as a positive feedback mechanism to facilitate trpm2 channel activation by non-cytolytic oxidative stress

As observed in WT microglial cells, exposure to H_2_O_2_ (10 μM) for 8 h induced PAR generation in TRPM2-KO microglial cells, which was also inhibited by treatment with PJ-34 (Figure 4(A)). Such results were anticipated, given that the TRPM2 channel activation takes place downstream of PARP or, more specifically, PARP-mediated ADPR generation. However, the PAR level in TRPM2-KO microglial cells was considerably less than that in WT microglial cells (Figure 4(A)), indicating that PARP generation also depends upon the TRPM2 channel.

Previous studies, as introduced above, have reported that intracellular Ca^2+^, originated from TRPM2 channel-mediated Ca^2+^ influx, can activate the PYK2/MEK/ERK pathway in monocytes to induce cytokine production [15] or act as a positive feedback mechanism by activating PARP and thereby mediating Zn^2+^-induced TRPM2-dependent microglial cell death [17]. It was interesting to test whether such a positive feedback mechanism is engaged in TRPM2 channel activation under non-cytolytic oxidative stress. H_2_O_2_-indcued PAR generation was strongly inhibited by treatment with 1 µM PF431396, a PYK2 inhibitor, or 3 µM U0126, an inhibitor of MEK that activates ERK (Figure 4(B,C)). Importantly, H_2_O_2_-induced TRPM2-mediated Ca^2+^ response was also strongly suppressed by treatment with the PYK2 or MEK/ERK inhibitors (Figure 4(D,E)). Collectively, these results suggest that the PYK2/MEK/ERK pathway acts as an important positive feedback mechanism to sustain the PARP activity and thereby facilitate TRPM2 channel activation.

### 3.5. The PKC/NOX and PYK2/MEK/ERK pathways are critically engaged in morphological changes of microglial cells induced by non-cytolytic oxidative stress

**Figure 5.**
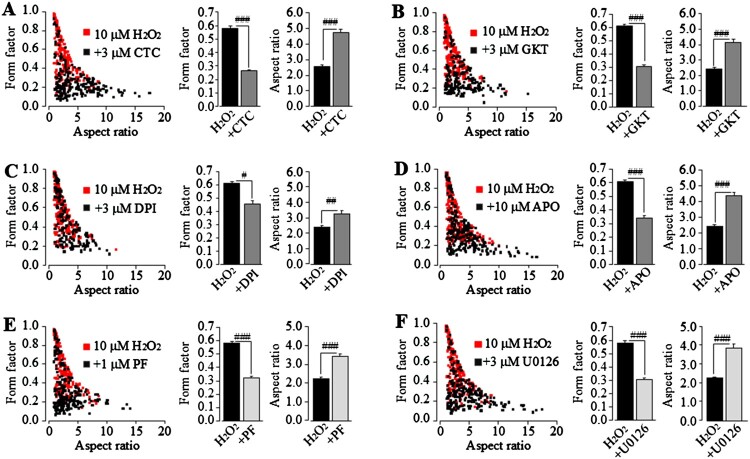
The PKC/NOX and PYK2/MEK/ERK signalling pathways are engaged in morphological changes induced by non-cytolytic oxidative stress in microglial cells. *Left*, Scatter plots showing the distribution of form factor and aspect ratio values for individual WT cells after exposure for 24 h to 10 µM H_2_O_2_. Cells were treated with 3 µM chelerythrine chloride (CTC)(A), 3 µM GKT137831 (GKT)(B), 3 µM DPI (C), 10 µM apocynin (APO) (D), 1 μM PF431396 (PF) (E), 3 µM U0126 (F) or DMSO as solvent control, 30 min prior to and duration exposure to H_2_O_2_; *Right*, mean values of form factor and aspect ratio in cells under indicated conditions. The mean values represent mean ± SEM of 3 average values from *N* = 3 independent experiments, with each average value from each independent experiment analysing 225 cells in 3 wells (75 cells per well) for every condition. #, *p* < 0.05; ##, *p* < 0.001; *p* < 0.005 compared to to H_2_O_2_-exposed cells prior treated with DMSO as solvent control.

We finally assessed the role of the PKC/NOX and PYK2/MEK/ERK pathways in morphological changes of microglial cells induced by non-cytolytic oxidative stress. The morphological changes induced by exposure to 10 μM H_2_O_2_ for 24 h were strongly suppressed by treatment with 3 μM CTC to inhibit PKC (Figure 5(A)), 3 μM GKT137831, 3 μM DPI or 3 μM apocynin to inhibit NOX (Fig.5B-D), 1 µM PF431396 to inhibit PYK2 (Figure 5(E)), or 3 µM U0126 to inhibit MEK/ERK (Figure 5(F)). These results indicate critical engagement of the PKC/NOX and PYK2/MEK/ERK pathways in non-cytolytic oxidative stress-induced TRPM2-dependent regulation of microglial cells.

## 4. Discussion

Researches over the past decade have revealed an important role of the TRPM2 channel in mediating oxidative stress-mediated cell death associated with multiple CNS damage and disease conditions [22,26]. However, under those conditions, oxidative stress induces microglial cells activation to elicit inflammatory response, which is more important, both physiologically and pathologically, than cell death. The findings of this study contribute to our evolving understanding of the TRPM2 channel in redox regulation of microglial cells.

The first contribution of this study is to enrich our understanding of the molecular and signalling mechanisms for TRPM2 channel activation by oxidative stress at levels inducing no cell death. As shown in this study, exposure to non-cytolytic oxidative stress induced by H_2_O_2_ can activate the TRPM2 channel or, more specifically, elicit TRPM2-mediated Ca^2+^ influx and subsequent increase in the [Ca^2+^], but requires extended exposure (Figure 2(A,B)). TRPM2-mediated Ca^2+^ responses were inhibited by TRPM2-KO (Figure 2(B)) or by treatment with 2-APB or PARP inhibitor PJ-34 (Figure 2(D)). Consistently, exposure to oxidative stress-induced PARP activation (Figure 4(A) and Fig.3S). These results have reinforced the important role of PARP activation in TRPM2 channel activation in response to oxidative stress [11,12]. As shown by previous studies, PKC and NOX can enhance oxidative stress by mediating ROS generation [17,24,25,27]. In this study, we demonstrated that inhibition of the PKC/NOX pathway attenuated ROS generation (Figure 3(A,B)) and PARP activation (Figure 3(C,D)), as well as TRPM2-mediated Ca^2+^ response (Figure 3(E,F)). These results support critical involvement of the PKC/NOX pathway, albeit the specific isoforms remaining to be defined, in ROS generation in initiating the activation of PARP and TRPM2 channel by non-cytolytic oxidative stress in microglial cells.

The second contribution of this study is to expand our understanding of the molecular and signalling mechanisms driving oxidative stress-induced activation of the TRPM2 channel and its potential role in redox regulation of microglial cell function. The PYK2/MEK/ERK pathway is known to regulate gene expression among other cellular processes that are involved in survival and inflammation [28,29]. As shown in the present study, H_2_O_2_-induced PAR generation (Figure 4(B,C)) and TRPM2 channel-mediated Ca^2+^ response (Figure 4(D,E)) were strongly reduced by inhibiting PYK2 or MEK/ERK, providing evidence to suggest the PYK2/MEK/ERK pathway as a positive feedback mechanism that facilitates or sustain the activation of PARP and TRPM2 channel. As anticipated, deletion of the TRPM2 expression ablated this positive feedback mechanism, leading to reduced PARP activation induced under the same level of oxidative stress (Figure 4(A)). Our previous study shows that such a signalling mechanism, namely, PKC/NOX-mediated ROS generation and activation of the PYK2/MEK/ERK signalling pathway, drives Zn^2+^-induced microglial cell death [17]. More often, microglial cells, as the major immunocompetent cells in the CNS, become activated from the homeostatic state in response to oxidative stress under a multitude of pathological factors. As observed in this study, exposure to non-cytolytic oxidative stress-induced salient changes in cell morphology, one of the distinct features accompanying or characteristic of microglial activation, with causing no cell death (Fig. 1S). Such morphological changes were consistently prevented by TRPM2-KO (Figure 1(A,C)) or treatment with 2-APB or PJ-34 (Figure 1(D)), indicating a critical role of the TRPM2 channel in microglial activation and, furthermore, were suppressed by inhibition of the PKC/NOX pathway and the PYK2/MEK/ERK pathway (Figure 5). These observations are consistent with the notion that oxidative stress can alter intracellular Ca^2+^ signalling [30,31] and that Ca^2+^ influx is a critical step in the transition of microglial cells from the homeostatic to activated state [32]. Collectively, these studies have expanded our understanding of the molecular and signalling mechanisms driving oxidative stress-induced activation of the TRPM2 channel and, moreover, its role in redox regulation of microglial cell function.

The study has limitations. It was confined to cell-based analysis and, the above-described mechanism remains to be testified in vivo. Neuroinflammation, due to low-level/grade but chronic microglial activation, plays a critical role in the progression of multiple CNS diseases and, efforts are required to evaluate the redox regulation of microglial functions such as generation of cytokines and, moreover, the significance of such microglial functions in neuroinflammation. In addition, treatment with 2-APB at 10 or 100 μM reduced H_2_O_2_-induced effects on microglial cells and these concentrations are higher than the potency of 2-APB reported to inhibit TRPM2 (1.2 μM) [33] and, due cautions should be exercised to interpret such data, albeit supporting the conclusion drawn from using TRPM2-KO microglial cells.

## 5. Summary

This study demonstrates the important role of PKC/NOX-mediated ROS generation and the PYK2/MEK signalling pathway as a positive feedback mechanism in driving TRPM2 channel activation and microglial activation in response to non-cytolytic oxidative stress. Future efforts for example using animal models are required to explore the significance of such a mechanism in the redox regulation of microglial cells in vivo and contribution in CNS damage and diseases.

## Ethical approval statement

Use of mice for microglial cell preparation in this study was prior approved by the University of Leeds Ethical Review Committee and conducted in accordance with the University of Leeds guidelines and procedure and conforming to the UK Home Office rules and regulations.

## Supplementary Material

Supplemental data.docx

## Data Availability

Data available within the article or its supplementary materials.
