# Supplementary material for: The PKC/NOX/ROS and PYK2/MEK/ERK/PARP signalling pathways drive TRPM2 channel activation induced by non-cytolytic oxidative stress in microglial cells
Source: Redox Rep. 2025 May 15;30(1):2503131. doi: 10.1080/13510002.2025.2503131 (PMC12086945; doi:10.1080/13510002.2025.2503131)
Supplement: Supplemental data.docx [file YRER_A_2503131_SM6681.docx]

**Supplemental data**

Sharifah Alawieyah Syed Mortadza ^2,3,*^, Nur Zulaikha Mohamad Zahir ^2^, Chew Tze Wei ^2^, Lin-Hua Jiang ^1,3*^


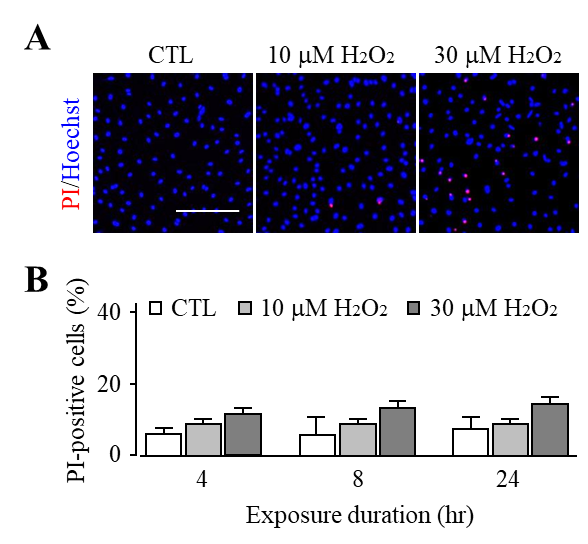


**Figure 1S Exposure to lower concentration H_2_O_2_ induces no microglial cell death.**

(a) Representative images showing microglial cell death (top row: PI-stained dead cells; bottom row: all cells stained with Hoechst) in WT cells upon exposure for 24 hr to 10-30 μM H_2_O_2_. (b) Summary of the mean percentage of PI-positive death cell under indicated conditions, representing mean ± SEM of three average values from 3 independent experiments (N = 3), with each average value from analysing 3 wells of cells (at least 75 cells per well and 225 cells in total) for every condition in each independent experiment.

**
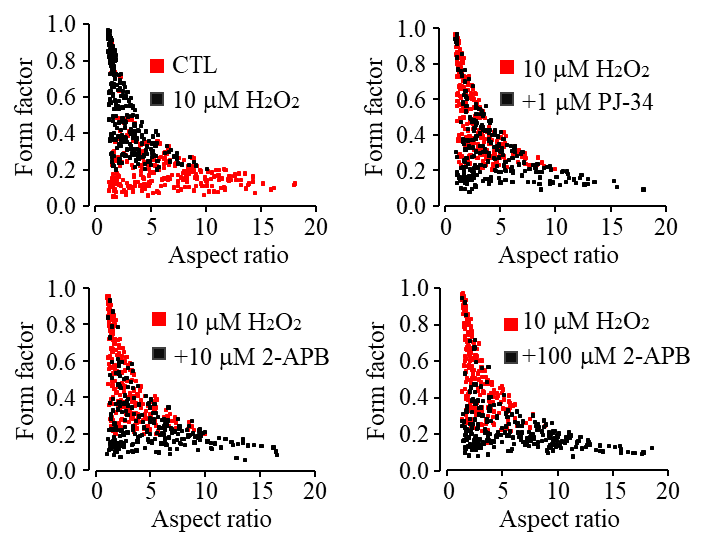
**

**Figure 2S. Morphological changes of microglial cells induced by exposure to non-cytolytic concentrations of H_2_O_2_ were inhibited by treatment with 2-APB and PJ-34.** Scatter plot showing distribution of form factor and aspect ratio of individual WT microglial cells after exposure to 10 µM H_2_O_2_ for 24 hours alone or, treatment with 10 and 100 μM 2-APB or 1 μM PJ-34, 30 min prior and duration exposure to H_2_O_2_.


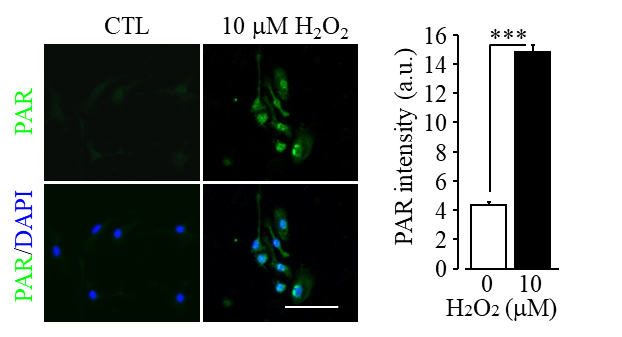


**Figure 3S. Exposure to non-cytolytic concentrations of H_2_O_2_ induces activation of PARP.** *Left*, Representative images showing PAR generation (top row: PAR; bottom row: co-staining with DAPI) in individual microglial cells after exposure for 8 hours to 10 µM H_2_O_2_. *Right*, mean H_2_O_2_-induced PAR generation in microglial cells under indicated conditions, representing mean ± SEM of three average values from 3 independent experiments (N = 3), with each average value from analysing 3 wells of cells (50 cells per well and 150 cells in total) for every condition in each independent experiment. Scale bar, 40 μm. ***, p < 0.005 compared to cells without exposure to H_2_O_2_.

**
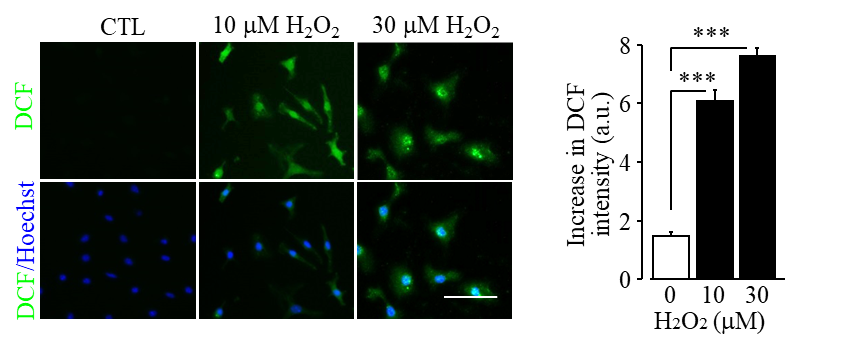
**

**Figure 3S. Exposure to non-cytolytic concentrations of H_2_O_2_ induces ROS generation in microglial cells.** *Left,* Representative fluorescent images showing the cellular ROS level (top row: DCF; bottom row: co-staining with Hoechst) in microglial cells without (CTL) and with exposure to H_2_O_2_ at indicated concentrations for 8 hours. *Right*, mean ROS generation in microglial cells, representing mean ± SEM of three average values from 3 independent experiments (N = 3), with each average value from analysing 3 wells of cells (50 cells per well and 150 cells in total) for every condition in each independent experiment. Scale bar, 40 µm. ***, p < 0.005 compared to cells without exposure to H_2_O_2_.
